# The commercially available STAT3 inhibitor 5,15-diphenylporphyrin (5,15-DPP) does not directly interact with STAT3 core residues 127–722

**DOI:** 10.1186/s13104-020-05189-w

**Published:** 2020-07-20

**Authors:** Siphokazi Sinethemba Mtwebana, Earl Prinsloo

**Affiliations:** Biotechnology Innovation Centre, Rhodes University, P.O. Box 94, Grahamstown, 6140 South Africa

**Keywords:** STAT3, Surface plasmon resonance, JAK-STAT3

## Abstract

**Objective:**

Target specific small molecule inhibitors has driven signaling pathway discovery and are used as common positive controls in drug discovery screens. During a biophysical screen, using surface plasmon resonance spectroscopy, of a novel small molecule library for the Signal Transducer and Activator of Transcription 3 Src Homology 2 (STAT3-SH2) low molecular weight interactors we evaluated commercial inhibitors S3I-201 and 5,15-diphenylporphyrin (5, 15-DPP) as positive controls.

**Results:**

Here, we show using surface plasmon resonance spectroscopy that a common STAT3-SH2 inhibitor, 5,15-diphenylporphyrin (5, 15-DPP), does not bind STAT3 core amino acid residues 127 to 722 relative to another commercially available SH2 inhibitor, S3I-201. This finding should provide caution in data interpretation when using 5,15-DPP in in vitro and in vivo laboratory investigations.

## Introduction

It is not uncommon to probe signaling pathways using specific small molecule inhibitors in order to elucidate key cascading events [1]. The deceptively simple Janus kinase-Signal Transducer and Activator of Transcription 3 (JAK-STAT3) signaling pathway has numerous commercially available inhibitors available targeting specific domains of the STAT3 protein [2–4]. The Src Homology 2 (SH2) domain is often targeted as a modulator of a key event in STAT3 activation, namely canonical phosphorylation of tyrosine 705 by Janus kinase 2 and subsequent nuclear targeting. Previously described inhibitors 2-Hydroxy-4-[[[[(4-methylphenyl)sulfonyl]oxy]acetyl]amino]-benzoic acid (NSC 74859) commonly termed S3I-201 and 5,15-diphenylporphyrin (5, 15-DPP) are typically described as SH2 specific inhibitors [5, 6].

Discovered through in silico screening, S3I-201 is a cell-permeable STAT3 inhibitor that binds to the STAT3-SH2 domain, prevents phosphorylation/activation, dimerization, and DNA-binding [5]. Similarly, the inhibitor 5,15-DPP has been described as a selective binder to STAT3 as an inhibitor of dimerization by interaction with the Src homology 2 (SH2) domain; reducing nuclear translocation and DNA binding [6].

## Main text

### Methods

Briefly, surface plasmon resonance (SPR) spectroscopy was performed using the ProteOn™XPR36 Interaction Array System (Bio-Rad) in 40 mM HEPES, pH 7.4, 150 mM KCl and 5 mM MgCl_2_. The sensor chips were initialized, primed, and preconditioned in horizontal and vertical parallel flow channels 0.5% (w/v) sodium dodecyl sulfate, 100 mM HCl and 0.85% (v/v) phosphoric acid at 30 μL/min. Recombinant mouse STAT3βtc (core residues 127–722, a kind donation from Dr Christoph W. Müller, EMBL Heidelberg) was expressed, purified [7, 8] and the conformation was assessed using Fourier Transformed Infrared Spectroscopy as previously described by our group [9]; STAT3 was immobilized between 4000 and 6000 RU using standard amine coupling in 20 mM sodium acetate buffer (pH 4) at 30 µl/min. Analyte injections of S3I-201 and 5,15-DPP were performed at 30, 60 and 100 µL/min for 60 s association and 600 s dissociations to observe interactions and mass transfer effects. Running buffer blank injections were performed following surface pulse regeneration (18 s) using 3 M guanidine HCl, 10 mM Tris–HCl, pH 8 at 100 µL/min. All data were collected using the ProteOn Manager (v3.1) and corrected by double referencing using the buffer blank injections and a blank ligand channel.

## Results and discussion

**Fig. 1:**
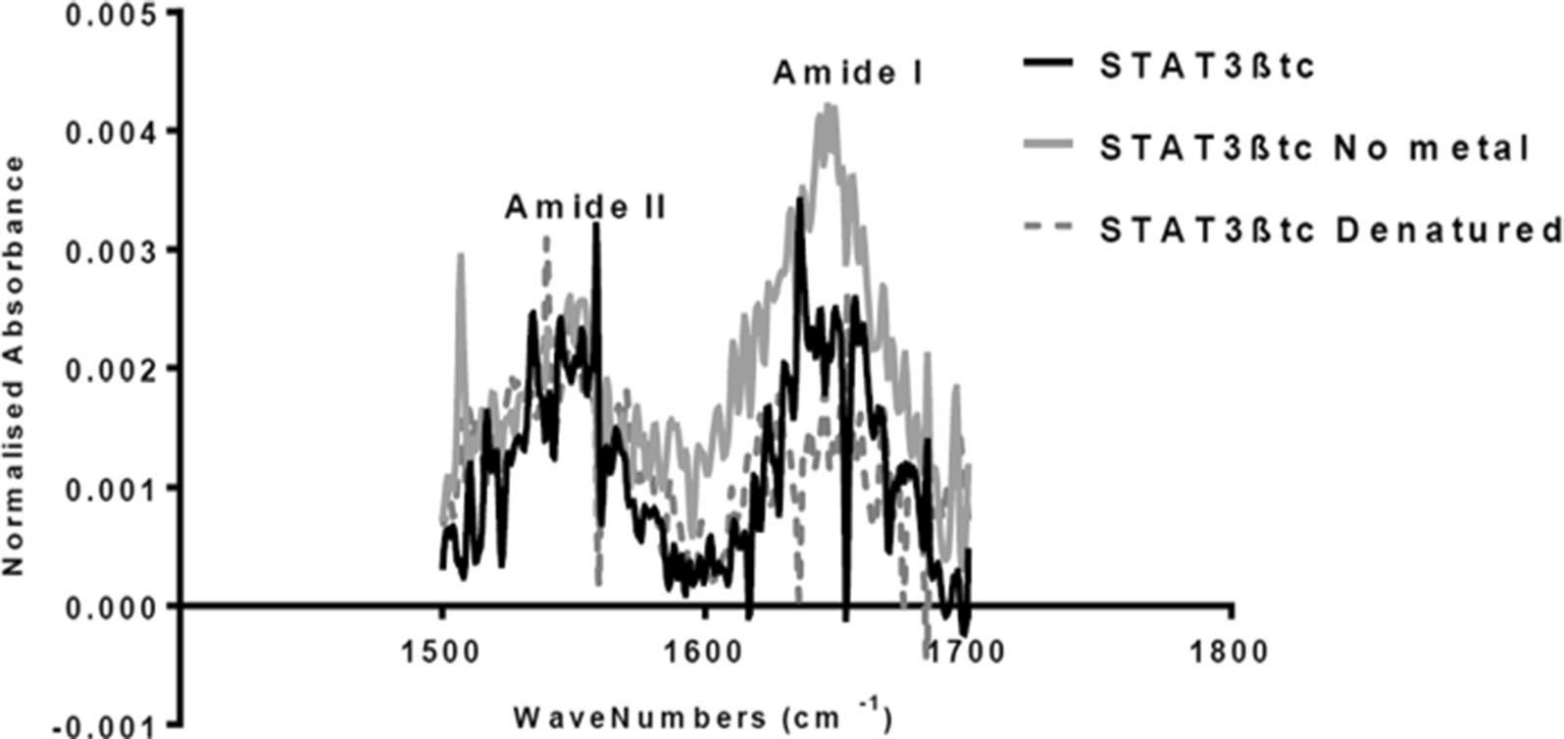
FTIR spectra for structural integrity of recombinant STAT3 (in presence and absence of Mg2 + containing buffer) and thermally denatured protein. Spectra represent Amide II (1500–1600 cm^−1^) and Amide I (1600–1700 cm^−1^). Divalent cations have been shown to promote homodimerization of STAT3 [10], this appears to have resulted in an altered Amide I peak

Structural analysis of purified recombinant STAT3 was performed using FTIR spectroscopy (Fig. 1) in the presence and absence of divalent magnesium compared to thermally denatured STAT3. The lack of amide I peak in the thermally denatured samples show that prolonged heating denatures all secondary structures relative to the native secondary structure of the monomer (no metal). As divalent cations have been shown to promote homodimerization of STAT3 [10], FTIR analysis in the the presence of magnesium showed an altered amide I peak relative to the monomeric and denatured samples. Becker et al. [7] describe the homodimerization as a function of the interacting SH2 domains; the pattern observed in Fig. 1 is likely a result of the interaction.

**Fig. 2:**
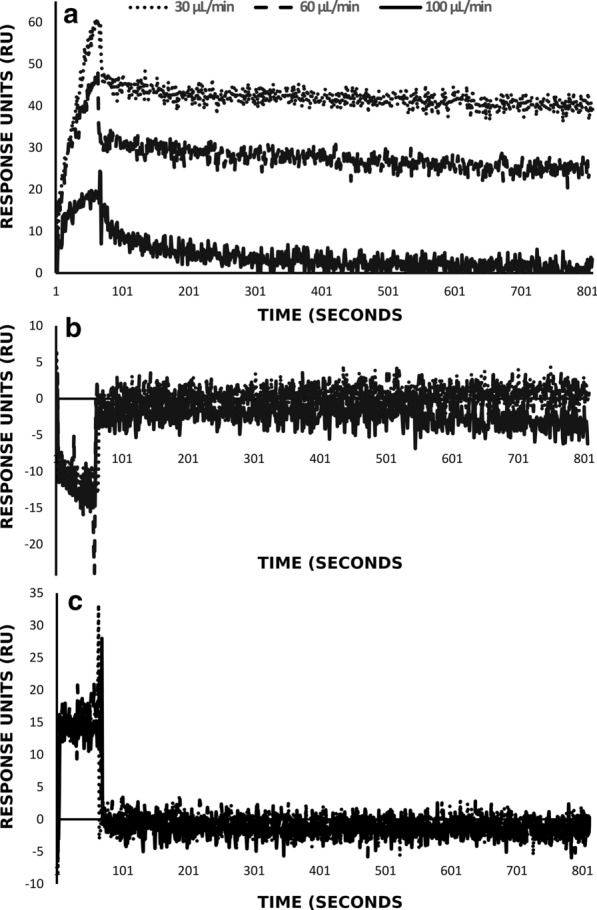
Sensorgrams showing comparative binding of immobilized recombinant STAT3 to commercial inhibitors (**a**) S3I-201 at 80 µM (**b**) 5, 15-DPP at 80 µM and (**c**) 5,15-DPP at 100 µM. Sensorgrams are representative of 3 flow rates as indicated

The sensorgrams shown in Fig. 2a are representative of the effect of increased flow rates (30–100 µL/min) on single concentrations of S3I-201 as analyte with monomeric STAT3 as ligand. The lower flow rates (30 and 60 µL/min) show defined mass transfer limited interactions with the association phase exhibiting linearity whereas the analyte at 100 µL/min shows a clear exponential association phase with the distinct logarithmic decay observable in the dissociation phase without the immediate bulk shift as is displayed by the analyte injections at 30 and 60 µL/min, respectively. This served to highlight a defined interaction event between S3I-201 and immobilized STAT3. The small molecule inhibitor 5,15 DPP failed to show any form of interaction (Fig. 2b) where flow rate and increased concentration (Fig. 2c) made little difference to a measurable response in both the association and dissociation phases. Despite the previous report of a high affinity direct interaction by Uehara et al. [6], no data was shown to support the claim of the K_D_ of 880 nM.

## Limitations

One caveat to the work shown here may be that 5,15 DPP may indeed bind STAT3 but not at the SH2 domain; the construct used lacks the first 126 amino acids of the N-terminal cooperative DNA binding domain required for STAT3 concatemerization at DNA binding sites and may play a role in transcriptional regulation [11]. Taken together with the data presented by Uehara et al. [6] i.e. that tyrosine phosphorylation is not inhibited and a decrease in nuclear localization and DNA binding is observed it may in fact be that 5,15-DPP interacts with regions in the N terminal of STAT3. Although it cannot be discounted that the observations of STAT3 inhibition by 5,15-DPP may be as a result of a positive off target effect. We do believe however, based on the data presented here taken together with the work of Uehara et al. [6], that 5,15-DPP should not be marketed as an SH2 inhibitor.

## Supplementary information

**Additional file 1.** Representative exported dataset of controls S3I-201 and 5,15-DPP from surface plasmon resonance spectroscopy screening experiment used to generate Fig. 2. Data for single concentration (80 μM) included are values collected at 30, 60 and 100 μL/min. Data for single concentration (100 μM) of 5, 15-DPP included at same flow rates.

## Data Availability

Raw data for the SPR data is provided as Additional file 1. The FTIR data is provided as processed data in the article.

## Abbreviations

STAT3: Signal transducer and activator of transcription 3
STAT3βtc: Signal transducer and activator of transcription 3 beta truncated
SH2: Src 2 Homology
JAK: Janus kinase
5, 15-DPP: 5,15-diphenylporphyrin
SPR: Surface plasmon resonance spectroscopy
RU: Response units
